# NKG2A gene variant predicts outcome of immunotherapy in AML and modulates the repertoire and function of NK cells

**DOI:** 10.1136/jitc-2023-007202

**Published:** 2023-08-30

**Authors:** Brwa Ali Hussein, Linnea Kristenson, Silvia Pesce, Anne Wöhr, Yarong Tian, Alexander Hallner, Mats Brune, Kristoffer Hellstrand, Ka-Wei Tang, Elin Bernson, Fredrik B Thorén

**Affiliations:** 1 TIMM Laboratory, Sahlgrenska Center for Cancer Research, University of Gothenburg, Gothenburg, Sweden; 2 Department of Medical Biochemistry and Cell Biology, Institute of Biomedicine, University of Gothenburg, Gothenburg, Sweden; 3 Dipartimento di Medicina Sperimentale, Università di Genova, Genoa, Italy; 4 Department of Infectious Diseases, Institute of Biomedicine, University of Gothenburg, Gothenburg, Sweden; 5 Department of Hematology, Institute of Medicine, University of Gothenburg, Gothenburg, Sweden; 6 Department of Obstetrics and Gynecology, Institute of Clinical Sciences, University of Gothenburg,Gothenburg, Gothenburg, Sweden

**Keywords:** Immunity, Innate, Killer Cells, Natural, Hematologic Neoplasms, Immunotherapy, Genetic Markers

## Abstract

**Background:**

The natural killer (NK) complex (NKC) harbors multiple genes such as KLRC1 (encoding NKG2A) and KLRK1 (encoding NKG2D) that are central to regulation of NK cell function. We aimed at determining to what extent NKC haplotypes impact on NK cell repertoire and function, and whether such gene variants impact on outcome of IL-2-based immunotherapy in acute myeloid leukemia (AML).

**Methods:**

Genotype status of NKG2D rs1049174 and NKG2A rs1983526 was determined using the TaqMan-Allelic discrimination approach. To dissect the impact of single nucloetide polymorphim (SNP) on NK cell function, we engineered the K562 cell line with CRISPR to be killed in a highly NKG2D-dependent fashion. NK cells were assayed for degranulation, intracellular cytokine production and cytotoxicity using flow cytometry.

**Results:**

In AML patients receiving immunotherapy, the NKG2A gene variant, rs1983526, was associated with superior leukemia-free survival and overall survival. We observed that superior NK degranulation from individuals with the high-cytotoxicity NKG2D variant was explained by presence of a larger, highly responsive NKG2A^+^ subset. Notably, NK cells from donors homozygous for a favorable allele encoding NKG2A mounted stronger cytokine responses when challenged with leukemic cells, and NK cells from AML patients with this genotype displayed higher accumulation of granzyme B during histamine dihydrochloride/IL-2 immunotherapy. Additionally, among AML patients, the NKG2A SNP defined a subset of patients with HLA-B-21 TT with a strikingly favorable outcome.

**Conclusions:**

The study results imply that a dimorphism in the NKG2A gene is associated with enhanced NK cell effector function and improved outcome of IL-2-based immunotherapy in AML.

**WHAT IS ALREADY KNOWN ON THIS TOPIC**

- Several studies have suggested that natural killer complex (NKC) locus haplotypes affect NK cell responses in cancer and most of these studies have assumed that these effects are related to variants of the gene encoding the activating receptor, NKG2D. However, there are other genes in NKC locus, and it is unclear how these genes influence NK cell function and clinical outcome in cancer.

**WHAT THIS STUDY ADDS**

- This study aimed at discerning how NKG2D and NKG2A gene variants may affect both the NK cell response and the clinical outcome of IL-2-based immunotherapy in acute myeloid leukemia (AML). We discovered that NKC gene variants were associated with improved survival in AML. Surprisingly, multivariate models that considered both NKG2A and NKG2D variants suggested that the NKG2A, rather than the NKG2D, was driving this survival benefit.

**HOW THIS STUDY MIGHT AFFECT RESEARCH, PRACTICE OR POLICY**

- Study findings suggest that the NKG2A rs1983526 gene variant may be of predictive value in immunotherapeutic interventions. Potentially, NKC locus gene variants could be an aspect to consider in donor selection for adoptive immunotherapy using NK cells for patients with cancer.

## Introduction

Acute myeloid leukemia (AML) is characterized by rapid proliferation and accumulation of immature myeloid cells in bone marrow and blood.^1–4^ Although most patients achieve complete remission (CR), a large proportion of non-transplanted patients will relapse within 1–2 years with a poor long-term outcome. There are several studies suggesting a central role for natural killer (NK) cells in AML,^5–11^ and stimulating NK cells to target residual leukemic cells is an attractive strategy to prevent relapse. In a phase III clinical trial (n=320), immunotherapy with histamine dihydrochloride (HDC) and interleukin-2 (IL-2) was found to prevent relapse in the post-consolidation phase of AML.^12 13^ Further studies have pinpointed that aspects of NK cell function impact favorably on the outcome of AML patients receiving HDC/IL-2.^14–18^

Regulation of NK cell function is governed by activating and inhibitory germline-encoded receptors. The activating receptors include the natural cytotoxicity receptors (NKp46, NKp44 and NKp30), DNAM-1 and NKG2D. NKG2D recognizes stress-induced proteins and contributes to NK cell-mediated cytotoxicity as well as costimulates cytokine production.^19 20^ The inhibitory receptors, such as killer immunoglobulin-like receptors (KIRs) and the CD94/NKG2A heterodimer (hereafter referred to as NKG2A), recognize HLA class I molecules leading to NK cell tolerance toward healthy autologous cells with intact HLA expression.^21^ The inhibitory receptors also calibrate the functional status of NK cells in a process known as education that refines NK cell responses toward target cells. While inhibitory KIRs recognize various specific HLA molecules, NKG2A interacts with the non-classical HLA-E that mainly present leader peptides from HLA-class I.^22^

In a seminal paper, Imai *et al* found a correlation between high natural cytotoxicity and lower incidence of cancer.^23^ Eight SNPs in the NK complex (NKC) region were reported to be associated with the altered natural cytotoxicity.^24^ Two NKC haplotypes were defined that are associated with high or low NK cell cytotoxicity and in turn with cancer incidence and outcome.^24–27^ Despite the pile of data suggesting associations between SNPs in the NKC locus and cancer risk, few studies have addressed functional consequences of these SNPs for NK cell function,^28^ and most studies have attributed the impact of the SNPs to be solely related to NKG2D despite scarcity of supporting data.^24^ On the other hand, it was reported that a dimorphism in the sequence encoding the leader peptide of HLA-B, rs1050458 (HLA-B-21), determines whether the peptide can be presented by HLA-E or not.^29 30^ This dichotomy has been proposed to influence the functional status of NKG2A^+^ cells and to be of clinical relevance in leukemia and HIV infection.^16 31–33^ It is not known, however, to what extent NKC locus SNPs regulate the interaction between NK cell receptors and their respective ligands.

Here, we set out to investigate the impact of NKC locus SNPs on NK cell function and to determine its relevance for the outcome of AML immunotherapy. We report that an NKG2A SNP impacts leukemia-free survival (LFS) and overall survival (OS) in AML patients receiving IL-2-based immunotherapy. Individuals with the favorable NKG2A genotype (GG) had a skewed NK cell repertoire toward NKG2A^+^ cells, mounted higher cytokine responses against leukemic cells, and NK cells from AML patients with this genotype displayed higher accumulation of granzyme B during HDC/IL-2 immunotherapy. We also observed that the favorable NKG2A genotype defined a subset of HLA-B-21 TT individuals with a strikingly superior outcome. Our data highlight the impact of NKG2A gene variants on NK cell function and clinical outcome of IL-2-based immunotherapy in AML.

## Materials, patients and methods

### Patients and healthy donors

Eighty-four AML patients aged 18–79 years in first CR who were not eligible for allogeneic stem cell transplantation were enrolled in a phase IV clinical trial (Re:Mission). Three patients withdrew consent; the other patients received 10 consecutive 3-week courses of HDC (0.5 mg, subcutaneously twice daily) and low-dose IL-2 (16 400 IU/kg, subcutaneously twice daily) over a period of 18 months. Two patients received allogeneic transplantation after relapse, but died within the follow-up period and were included as events in the OS analysis. Results for primary end points and patient characteristics can be found in references 14 15 34 35. Patient samples were collected before and after the first treatment cycle. Healthy donor peripheral blood mononuclear cells (PBMCs) were isolated using density gradient centrifugation with Lymphoprep (Stemcell Technologies). NK cells were generated by negative selection using MACS NK isolation kit (Miltenyi Biotec). To generate polyclonally activated NK cells, NK cells were cultured with irradiated 221G and allogeneic PBMCs in complete medium supplemented with 600 IU/mL IL-2 (Chiron) and 5 µg/mL phytohemagglutinin (PHA-M, Sigma). After 5 days, the medium was gradually replaced with the same medium without PHA-M.

### Genotyping

DNA was extracted from blood cells using the Qiagen DNeasy Blood & Tissue kit. NKG2D rs1049174 and NKG2A rs1983526, hereafter referred to as NKG2D SNP and NKG2A SNP, respectively, were genotyped using the TaqMan-Allelic discrimination technique. The Re:Mission trial patients’ HLA-B-21, rs1050458, genotypes were determined using a One Lambda SSO typing kit as described in.^16^ HLA-B-21 rs1050458 in healthy donors was genotyped using an agarose gel electrophoresis method.^36^

### CRISPR-Cas9-editing of K562 cells

CRISPR-Cas9 was used to knockout (KO) the genes encoding B7-H6, PVR and NECTIN-2 in K562 cells (CCL-2243, ATCC). In brief, lentiviral particles produced from lentiCas9-Blast (Addgene plasmid #52962), deposited by Feng Zhang lab, were used for transduction of K562 cells by spinoculation in the presence of 5 µg/mL polybrene. After a 2-day recovery, Cas9-expressing cells were selected by culture in the presence of blasticidin (30 µg/mL, 10 days).

For KO of ligand genes, a general gRNA insert was generated to contain the scaffold tracrRNA sequence and allow incorporation into a plasmid and ligation of gene-specific crRNAs (online supplemental table 1). Inserts and plasmids mEGFP-C1 and EBFP2-C1 (plasmids #54759 and # 54665, deposited to Addgene by Michael Davidson) were digested using restriction enzymes FastDigest BamHI and FastDigest Xhol (Thermo Fischer Scientific). Gel purification (TaKaRa RECOCHIP) was performed prior to ligation with T4 DNA ligase (New England Biolabs) and plasmid amplification by DH5α electroMAX (Invitrogen).

10.1136/jitc-2023-007202.supp1 Supplementary data

The crRNAs were chosen from validated gRNAs by Broad institute and confirmed using Synthego’s verification tool and were ordered from IDT with overhangs for restriction enzyme BbsI and 5’ phosphorylation. The oligonucleotides were annealed before combined digestion/ligation using BbsI-HF (New England Biolabs) and T4 DNA ligase. Transfection was performed using Neon transfection system (100 µL tips, 1450V, 10 ms, 3 pulses: Thermo Fisher Scientific). Single cell sorts were performed based on high GFP and/or BFP expression. After generation of clones, cells were stained with fluorochrome-conjugated antibodies to identify KO clones by flow cytometry. The deletion was then verified using Sanger sequencing (Eurofins).

### Degranulation, cytotoxicity, and intracellular staining assays

In degranulation assays, activated healthy donor NK cells (10 ng/mL IL-15, Peprotech or 500 IU/mL IL-2, Chiron) were cocultured with wild type (wt) or triple KO (tKO) K562 at a ratio of 1:1. In indicated experiments, NK cells were preincubated with 5 mg/mL of an NKG2D-blocking antibody. After 4 hours of incubation, cells were stained with the master mix of antibodies (online supplemental table 2). For cytotoxicity, NK cells were incubated with targets and dead cells were identified using Live/Dead or Topro3 staining. In some experiments, blocking antibodies to NCRs, DNAM-1 and NKG2D were used. In intracellular cytokine assays, PBMCs were stimulated overnight with IL-2 then cultured with wt-K562 cells for 5 hours in the presence of Brefeldin A. Afterwards, PBMCs were stained with a mix of antibodies (online supplemental table 2) followed by fixation and permeabilization (BD cytofix/cytoperm, BD Bioscience). Subsequently, PBMCs were stained for IFNg before flow cytometry analysis.

### RT-qPCR

One hundred K562 cells were sorted into each well in a 96 well containing 5 µL water with 1 mg/mL BSA (Thermo Fisher Scientific). Reverse transcription was performed using GrandScript cDNA Synthesis Kit (TATAA Biocenter) on 5 µL cell lysate in 50 µL reaction containing 1× TATAA GrandScript RT Reaction Mix and 1× TATAA GrandScript RT Enzyme. Reverse transcription was performed at 22°C for 5 min, 42°C for 30 min and 85°C for 5 min. All samples were diluted with nuclease-free water. Quantitative PCR was performed using SYBR GrandMaster Mix (TATAA Biocenter) with 2 µL preamplified cDNA in a 6 µL reaction containing 1× SYBR GrandMaster Mix and 400 nM of each primer (online supplemental table 1). A CFX384 Touch Real‐Time PCR System (Bio‐Rad) was used at 95°C for 2 min followed by 49 cycles of amplification at 95°C for 5 s, 60°C for 20 s and 70°C for 20 s followed by melting curve analysis. Detection of genomic DNA was assessed by including reverse transcription negative samples. Cycle of quantification values was determined by the second derivative maximum method using the CFX Manager Software V.3.1.

### Statistics

Statistical analysis was performed using GraphPad prism V.7.0. Unpaired two-sided Student’s t-test was applied to compare two groups. One-way analysis of variance, followed by Bonferroni’s multiple-comparison test, was performed for multiple group comparisons. Simple linear regression was used to show the relationship between two variables. Statistical analysis of OS and LFS was implemented using the log rank test. Variables that significantly predicted LFS and/or OS were further analyzed by univariable and multivariable cox regression analysis.

## Results

### AML patients carrying NKG2A rs1983526 GG show favorable outcome after HDC/IL-2 immunotherapy

**Figure 1:**
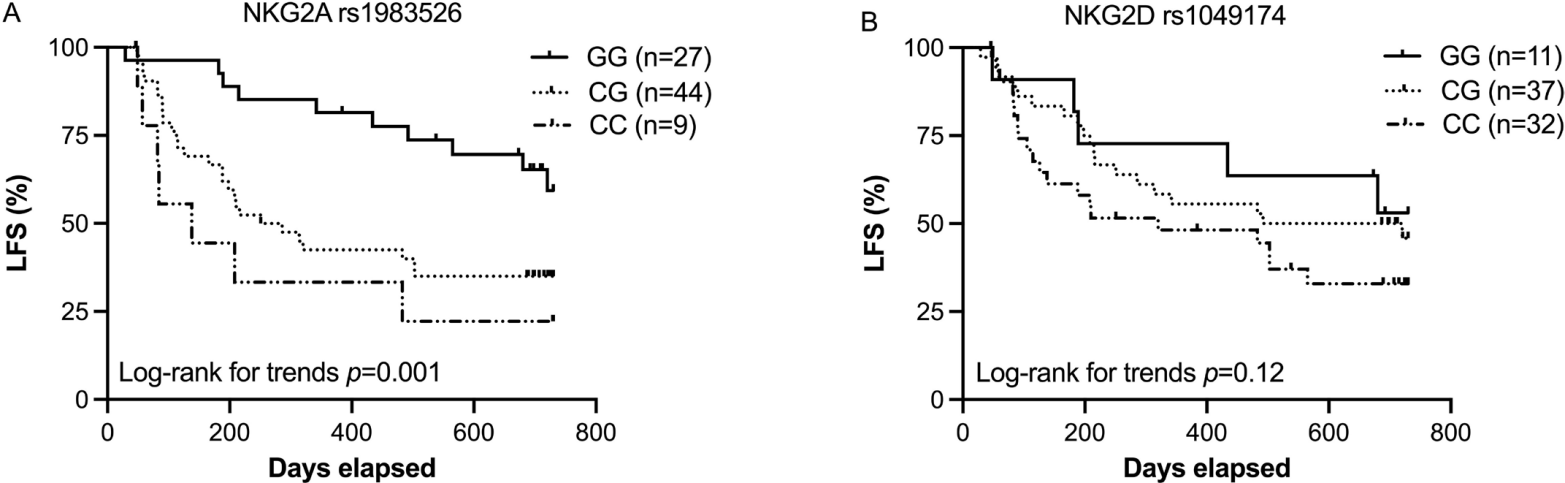
Impact of NKG2A and NKG2D gene variants on outcome of immunotherapy in AML. (A) Leukemia-free survival of AML patients carrying NKG2A (rs1983526) GG (n=27), CG (n=44) or CC (n=9), after receiving HDC/IL-2 treatment. (B) Leukemia-free survival of AML patients based on NKG2D (rs1049174) GG (n=11), CG (n=37) or CC (n=32), after receiving HDC/IL-2 treatment. AML, acute myeloid leukemia; HDC, histamine dihydrochloride; LFS, leukemia-free survival.

The variant, rs1049174, of the *KLRK1* gene, which encodes the activating receptor NKG2D, has been linked to improved anticancer immunity.^18 24^ However, this gene is located in close proximity to the *KLRC1* gene, which encodes NKG2A—another key regulatory receptor of NK cells and T cells. The NKG2D SNP, rs1049174, is thus in linkage disequilibrium with the NKG2A SNP rs1983526. We set out to investigate to what extent these NKC locus variants impacted on the incidence of relapse and death among AML patients receiving HDC/IL-2 immunotherapy. To this end, all patients were typed for both rs1049174 and rs1983526. As shown in figure 1A, patients carrying the NKG2A rs1983526 G allele showed superior LFS (p=0.001, log rank for trend) and OS (p=0.02, (online supplemental figure 1A). The NKG2D rs1049174 G allele also trended to be associated with OS (p=0.05 (online supplemental figure 1B) and LFS (p=0.12, figure 1B)). To further define the relative contribution by the two SNPs in a combined model, we performed Cox regression analysis, and found that the observed impact on LFS was associated with the NKG2A rs1983526 (p=0.01) and not the NKG2D rs1049174 (p=0.8). Furthermore, in a multivariable analysis, which included age, number of induction cycles, CMV serostatus and HLA-B rs1050458 genotype as covariates, NKG2A rs1983526 independently predicted LFS (table 1).

**Table 1:** Multivariable analyses of variables impacting on LFS

Gene variants	Univariate analysis			Covariate analysis (NKG2A and NKG2D SNPs in one model)			Multivariate analysis		
	HR	95% CI	P value	HR	95% CI	P value	HR	95% CI	P value
NKG2D SNP LFS	1.58	0.87 to 2.87	0.1	1.09	0.57 to 2.07	0.8	1.39	0.76 to 2.56	0.3
NKG2A SNP LFS	2.70	1.32 to 5.49	0.006	2.6	1.21 to 5.57	0.01	2.19	1.05 to 4.59	0.01

Cox regression analysis with both indicated SNPs as numerical predictors. In the covariate analysis, both SNPs were included in the same model. For the multivariate analysis, previously reported predictive variables affecting the clinical outcome of AML patients including age, risk group (ELN-2010), number of induction chemotherapy cycles to attain complete remission (1 or >1), number of consolidation courses (0–2 or>2), CMV serostatus and HLA-B-21 (rs1050458) genotype were analyzed by univariable Cox regression analysis. Covariates with p values of below 0.1 (age, number of induction chemotherapies cycles, CMV status and HLA-B-21 (rs1050458) genotype status, NKG2A SNP (rs1983526), NKG2D SNP (rs1049174)) were included in the multivariate analysis. The ELN-2010 risk stratification used in this study included various cytogenetic and molecular aberrations which stratify patients into favorable, intermediate I, intermediate II and adverse groups.^54^

AML, acute myeloid leukemia; CMV, cytomegalovirus; LFS, leukemia-free survival; SNP, single nucleotide polymoprhism.

### An NK cell degranulation response among NKG2D G/x donors is driven by an abundant NKG2A^+^ subset

The finding that NKG2A variants rather than NKG2D SNP determined the outcome of IL-2-based immunotherapy incited us to test the impact of the prototypic NKG2D SNP rs1049174 in functional studies. In the first set of experiments, we exposed healthy donor NK cells to the HLA-deficient K562 cell line. We did not observe differences in degranulation response between NK cells from individuals with at least one high-cytotoxicity NKG2D allele (rs1049174 G/x) and cells from individuals with two low-cytotoxicity alleles C/C (online supplemental figure 2A). However, NK cell killing is achieved by activating NK cell receptors acting in concert with one or a few dominant receptors, and we speculated that the lack of difference in NK cell degranulation might be due to NKG2D only playing a minor role in NK cell recognition of K562 cells. Using RT-PCR, we determined presence of NK cell receptor ligand transcripts in K562 cells. Transcripts for most NK cell activating receptor ligands were found in K562 cells (data not shown), and staining using antibodies or soluble receptor fusion proteins revealed clear ligand expression of B7-H6, PVR, NECTIN-2, ULBP1, ULBP2/5/6 and ULBP4, along with weaker expression of MICA/B (online supplemental figure 2B). Cytotoxicity experiments with blocking antibodies confirmed previous reports demonstrating a dominant role for NKp30.^37 38^ Notably, NKG2D blockade did only have a minor impact on NK cell cytotoxicity, which may explain why NKG2D variants did not affect NK cell responses to K562 cells (online supplemental figure 2C). However, by combining NKp30 blockade with other blocking mAbs, we observed that additional blockade of DNAM-1 or NKG2D offered further protection of K562 cells from NK cell lysis (online supplemental figure 2D).

**Figure 2:**
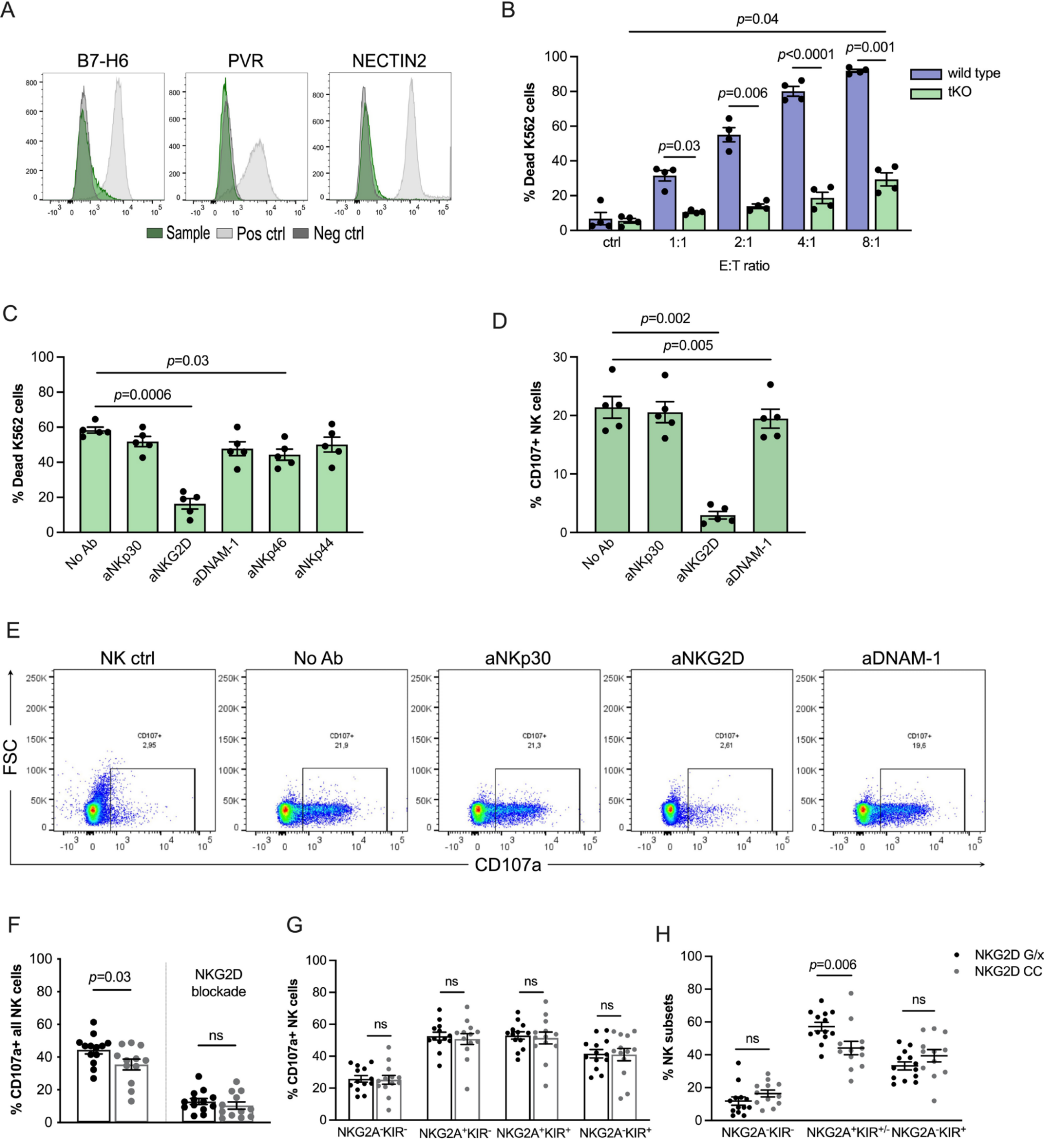
NK cell function based on NKG2D genotypes using an NKG2D-dependent model. (A) Flow cytometry staining of triple KO (tKO) K562 cells showing successful knockout of ligand genes. (B) Cytotoxicity assay of healthy donor resting NK cells against wt K562 and tKO K562 cells at various effector:target (E/T) ratios (n=4). (C) Cytotoxicity assay of polyclonally activated NK cells from healthy donors against triple knockout K562 at 8:1 E/T ratio, in the presence or absence of indicated NK cell receptor blockade (n=5). (D, E) Degranulation assay of healthy donor resting NK cells against tKO K562 combined with NK cell receptor blockade (n=5) with representative dot plots. (F) Degranulation assay of CD56^+^NK cells from healthy donors with NKG2D G/x or CC, preactivated with IL-15 overnight and cocultured on next day with tKO K562 for 4 hours in the presence or absence of NKG2D blocking antibody (G/x n=13 and CC n=12). (G) Degranulation response in different NKG2A^+/−^ and KIR^+/−^ NK cell subsets in healthy donors with G/x (n=13) or CC (n=12) genotypes. (H) Frequency of different NK cell subsets in healthy donors according to NKG2D SNP G/x (n=13) and CC (n=12). Error bars represent SEM. NK, natural killer; SNP, single nucleotide polymorphism.

Next, we used CRISPR to delete genes encoding the identified key ligands in K562 cells to further decipher the role of NKG2D for NK cell cytotoxicity. Double strand deletion of NCR3LG1 (encoding B7-H6), PVR and Nectin-2 was confirmed using Sanger sequencing, and antibody staining confirmed abrogated expression of targeted ligands in a triple-KO K562 cell line (NCR3LG1^−/−^PVR^−/−^Nectin-2^−/−^; hereafter referred to as tKO K562; figure 2A). As expected, the tKO K562 cell line displayed reduced sensitivity to NK cell lysis. However, at higher E/T ratios, tKO K562 cells were readily killed by NK cells (figure 2B). Notably, NK cell cytotoxicity and degranulation against tKO K562 cells were largely prevented by addition of the NKG2D blocking antibody BAT221, supporting that the tKO K562 model was relevant to address the role of NKG2D in NK cell regulation (figure 2C–E).

We then compared degranulation response of stimulated NK cells from donors with defined NKG2D rs1049174 genotypes toward tKO K562 cells. As expected, NKG2D receptor blockade abrogated NK cell degranulation. NK cells from donors carrying NKG2D G/x degranulated more efficiently toward tKO K562 cells than CC donors, but the difference was modest (figure 2F). In more detailed analyses, we determined the degranulation responses in subsets of NK cells based on the expression of the key inhibitory receptors, NKG2A and KIRs. As seen in figure 2G, the difference in degranulation response between G/x and C/C individuals was not due to enhanced degranulation by a specific subset of NK cells. Instead, G/x donors were found to harbor a significantly higher fraction of NKG2A^+^ cells when compared with CC individuals, and this subset degranulated more regardless of NKG2D genotype (figure 2H). Thus, the higher degranulation observed in NKG2D rs1049174 G/x donors was driven by a more abundant NKG2A^+^ population that responded more vigorously toward tKO K562 cells.

### Elevated expression of NKG2A and granzyme B among NKG2A GG AML patients receiving relapse-preventive immunotherapy

**Figure 3:**
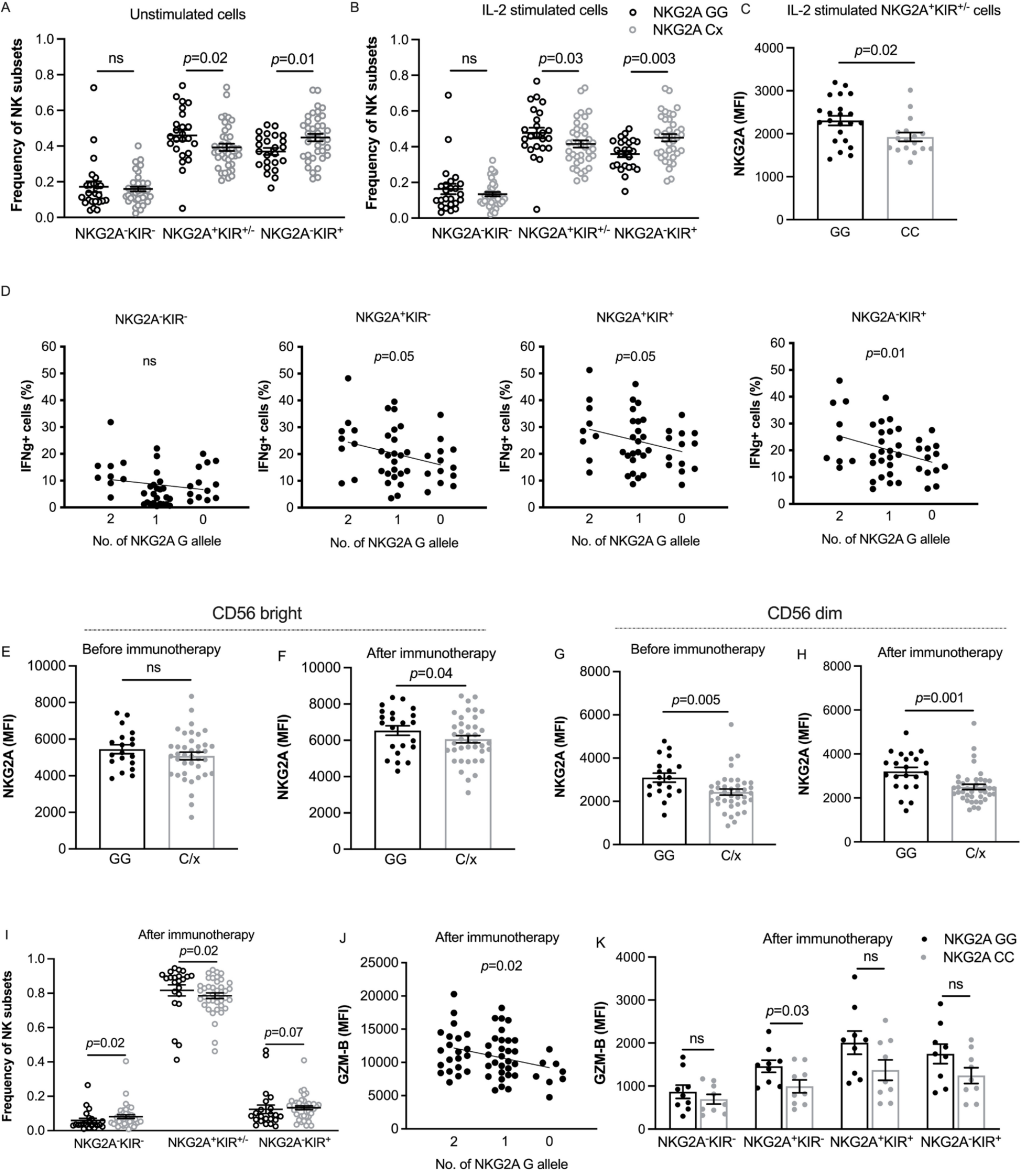
Subset distribution, IFNg response and granzyme B content in CD56^dim^ NK cells according to NKG2A genotypes. (A, B) NK cell subset distribution among NKG2A GG (n=24) or C/x (n=39) in healthy donor CD56^dim^ NK cells before and after cytokine stimulation. (C) Median fluorescence intensity of NKG2A expression in CD56^dim^ NK cells based on NKG2A SNP status (GG n=22 and CC n=17) in healthy donors after IL-2 stimulation. (D) Percentage of IFNg^+^ cells in IL-2-stimulated healthy donor CD56^dim^ NK cell subsets in indicated NKG2A SNP genotypes (GG n=9, CG n=23 and CC n=13) after coincubation with wt K562 cells. (E–H) Median fluorescence intensity (MFI) of NKG2A in NKG2A^+^ cells according to NKG2A SNP in CD56^bright^ NK cells (GG n=19 and C/x n=39) at the start of HDC/IL-2 immunotherapy (E) and after one course of treatment (GG n=22 and C/x n=40) (F); and CD16^+^ CD56^dim^ NK cells (GG n=19 and C/x n=39) at the start of immunotherapy (G) and after one course of immunotherapy (GG n=22 and C/x n=40) (H). (I) Frequency of NK subsets among CD56^dim^ NK cells of AML patients based on NKG2A SNP GG (n=22) and C/x (n=40) at the end of first course of HDC/IL-2 therapy. (J) Impact of NKG2A rs1983526 alleles on granzyme-B (GZM-B) content in CD16^+^ CD56^dim^ NK cells from AML patients after one course of immunotherapy (GG n=21, CG n=29 and CC n=8). (K) Granzyme-B expression in subsets of CD56^dim^ NK cells in AML patients carrying either NKG2A GG (n=9) or CC (n=9) after one course of immunotherapy. Error bars represent SEM. AML, acute myeloid leukemia; NK, natural killer; SNP, single nucleotide polymorphism.

In further experiments, we sought to address the reasons for the superior outcome of patients with the NKG2A GG genotype. First, we stained a cohort of primary AML blasts and healthy donor PBMCs for HLA-ABC and HLA-E. In accordance with previous studies,^16^ we found AML blasts to express normal levels of HLA-ABC, while the expression of HLA-E was significantly lower (online supplemental figure 3A,B). Next, we genotyped healthy donors at rs1983526 in the NKG2A-encoding gene KLRC1 and determined the effect of the gene variants on NKG2A expression and subset distribution among CD56^dim^ NK cells (gating strategy shown in online supplemental figure 4). We observed that donors with the favorable NKG2A genotype (GG) harbored a significantly higher fraction of NKG2A^+^ CD56^dim^ NK cells than C/x donors both before-and-after over-night activation with IL-2 (figure 3A,B). Furthermore, NKG2A^+^ NK cells from GG donors expressed significantly higher levels of NKG2A than CC donors after IL-2 stimulation (figure 3C). To examine whether the NKG2A rs1983526 impacted NK cell effector function, PBMCs, prestimulated overnight with IL-2, were coincubated with wt-K562 cells. After 5 hours, NK cell subsets were assayed for accumulation of intracellular IFNg. Interestingly, NKG2A GG donors displayed enhanced IFNg accumulation, and there was a clear gene dose effect as each C allele reduced the percentage of IFNg-producing NK cells. Notably, the superior response by NK cells from GG donors was not restricted to NKG2A^+^ subsets but was also observed among more differentiated KIR^+^NKG2A^−^ NK cells (figure 3D).

We next investigated whether similar patterns were present in the AML patients receiving HDC/IL-2 immunotherapy. Before the start of immunotherapy, NKG2A GG donors had higher expression levels of NKG2A in their NKG2A^+^ CD56^dim^ NK cells and this difference was further pronounced after one course of immunotherapy. Also, CD56^bright^ NK cells from patients with the NKG2A GG genotype displayed significantly higher expression of NKG2A after one course of immunotherapy (figure 3E–H). As previously reported, most NK cells in AML patients in remission expressed NKG2A.^16^ However, NKG2A GG patients still harbored a higher percentage of NKG2A^+^KIR^+/-^ cells when compared with C/x patients (figure 3I). Interestingly, CD56^dim^ NK cells from GG patients displayed significantly higher granzyme B expression after the first course of immunotherapy, and each C allele reduced the granzyme B content (figure 3J), indicating that the NKG2A G allele is associated with a higher NK cell cytolytic function. In accordance with the data obtained in the healthy donors, the elevated expression of granzyme B in NKG2A GG patients was not restricted to a certain NKG2A/KIR subset (figure 3K).

### SNPs at NKG2A and HLA-B-21 define a high-risk population among AML patients

**Figure 4:**
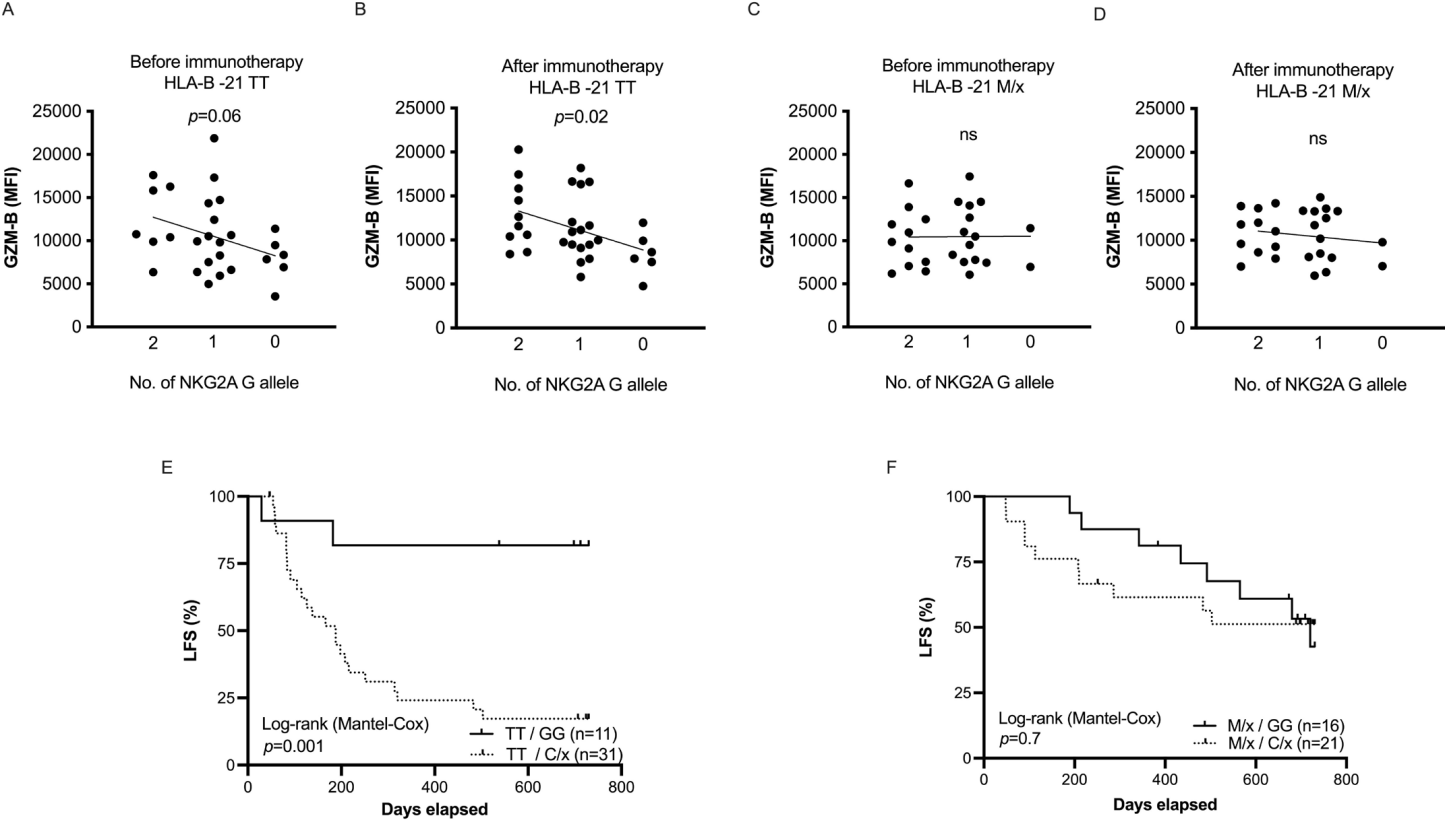
Combinatory effect of HLA-B-21 dimorphism and NKG2A gene variants on granzyme B levels and clinical outcome of IL-2 based therapy in AML patients. (A–D) Impact of NKG2A SNP alleles on granzyme-B load of CD16^+^ CD56^dim^ NK cells in AML patients at the beginning and after one course of IL-2 based therapy in HLA-B-21 TT carriers (A, B) and HLA-B-21 M/x carriers (C, D). (E) Leukemia-free survival for patients with NKG2A GG (n=11) or C/x (n=31) in patients with an HLA-B –21 TT genotype. (F) Leukemia-free survival for HLA-B –21 M/x patients dichotomized into NKG2A GG (n=16) and C/x (n=21). AML, acute myeloid leukemia; LFS, leukemia-free survival; NK, natural killer.

It was previously reported that a dimorphism at position -21 in HLA-B determines the relative role of KIRs and NKG2A in NK cell regulation.^31^ The leader sequence encoded by the HLA-B-21 M allele generates a high-affinity peptide that is presented on HLA-E 29 33 [Bibr R29], and its presence is associated with more functional NKG2A^+^ cells and superior outcome in AML.^16^ To clarify whether the impact of the NKG2A SNP on NK cell function and clinical outcome differed between HLA-B-21 genotypes, we stratified our patients based on the HLA-B-21 SNP and assessed how granzyme B content differed among genotypes. As shown in figure 4A,B, each additional NKG2A C allele reduced the intracellular content of granzyme B in HLA-B-21 TT individuals, whereas the granzyme B levels were unaffected by the NKG2A genotype in HLA-B-21 M/x individuals (figure 4C,D). In line with these results, no significant impact of the NKG2A SNP on the outcome of patients with the favorable HLA-B-21 M/x phenotype was observed. However, in patients carrying the otherwise unfavorable HLA-B-21 TT, the NKG2A GG genotype identified a population of patients with strikingly superior survival (figure 4E,F).

## Discussion

Several studies have demonstrated a link between NKC locus haplotypes and NK cell responses in cancer and most studies have presumed that these effects are related to the gene encoding NKG2D.^18 23 24 27 28^ However, there are additional genes in this locus that affect NK cell function, such as *KLRC1*, *KLRC2* and *KLRD2*, which encode NKG2A, NKG2C and CD94, respectively. The purpose of this study was to define the potential impact of NKG2D and NKG2A gene variants on NK cell function and outcome of HDC/IL-2 immunotherapy in AML. In summary, we found that NKC gene variants were associated with improved LFS after HDC/IL-2 immunotherapy, but surprisingly, multivariate models taking both NKG2A and NKG2D variants into consideration suggested that the survival benefit was driven by the inhibitory receptor NKG2A rather than the activating receptor NKG2D.

We also addressed the impact of the NKG2D variant in in vitro studies by genetic engineering of the prototypic NK cell target cell line, K562. By knocking out ligands to DNAM-1 and the NKp30 ligand, B7-H6, we skewed the receptor dependence for NK cell interactions with K562 cells and created a relatively resistant K562 cell line. However, at higher E/T ratios, these tKO K562 cells were readily killed by NK cells in a highly NKG2D-dependent manner, which allowed interrogating the impact of NKG2D SNPs on NKG2D function. Surprisingly, we only observed a small increase in degranulation response against the tKO K562 cells in NK cells from blood donors with the high cytotoxicity NKG2D allele. Furthermore, a more thorough subset analysis revealed that the slightly enhanced degranulation observed was driven by a larger subset of NKG2A^+^ cells. In accordance with this finding, we found individuals homozygous for the favorable allele of NKG2A SNP to harbor a higher fraction of NKG2A^+^ cells and fewer NKG2A^−^KIR^+^ NK cells. This may contribute to the improved outcome for this group of AML patients, as we observed that AML blasts express low levels of the inhibitory ligand HLA-E, but normal levels of HLA-ABC. Thus NKG2A^+^ cells may be able to mount a missing self-response against leukemic blasts, while KIR^+^ subsets are inhibited by the intact expression of other HLA molecules.

According to the rheostat education model, the functional status of NK cells is set by the inhibitory input under homeostatic conditions.^39^ Since the NKG2A GG was associated with higher expression of NKG2A, this model predicts that the increased steady-state inhibitory input would in turn generate more functional NKG2A^+^ cells in GG donors. NK cells from GG donors displayed a higher IFNg response after coincubation with K562 cells, but notably, the enhanced response was not restricted to NKG2A^+^ cells as also KIR^+^NKG2A^-^ NK cells from GG donors displayed elevated IFNg responses. Similarly, patients with NKG2A GG in our cohort presented with higher intracellular stores of granzyme B, and a more thorough analysis of subsets indicated presence of higher granzyme B levels across all NK cell subsets. Granzyme B stores have been proposed as a surrogate marker for the educational status of NK cells,^40^ and thus, our findings seem to violate the rules of the rheostat model. Taken together, these findings suggest that there are other hitherto unknown mechanisms, besides education, that may contribute to the high granzyme B phenotype and the elevated IFNg responses in NKG2A GG individuals. An interesting possibility is that enhanced NKG2A-signaling in NK cells from GG individuals results in epigenetic and/or metabolic changes that remain beyond NKG2A downregulation and KIR acquisition and account for the accumulation of granzyme B and elevated effector functions observed in this study. A more thorough understanding of these mechanisms may be important to develop efficient NK cell-based immunotherapies.

Enhanced NKG2A signaling may also be present in individuals with an HLA-B-21 genotype resulting in more HLA-E-presentable peptides and elevated HLA-E expression.^16 31^ However, a recent study suggests that these HLA-B-21 peptides outcompete other peptides and bind to HLA-E to generate an NKG2A antagonist/partial agonist.^33^ We did not observe any impact on outcome based on the NKG2A SNP in AML patients with HLA-B-21 M/x. However, the NKG2A GG genotype significantly improved LFS in patients with the unfavorable HLA-B-21 TT genotype. Furthermore, only in HLA-B-21 TT individuals did the NKG2A G allele confer upregulation of intracellular granzyme B. One interpretation is that the lack of impact of NKG2A GG genotype in M/x patients could reflect that their NK cells already receive sufficient inhibitory signaling via NKG2A due to the higher HLA-E expression levels that accompany the M/x genotype. Alternatively, in light of the study of Carrington and colleagues,^33^ M/x patients may have a larger fraction of antagonistic HLA-E, and thus only TT individuals will become more educated by the enhanced NKG2A expression. A complicating factor is also that NKG2A is upregulated on activated cells. Thus, elevated missing-self responses from NKG2A-expressing cells can either be a result of high education or reflect superior responses from preactivated cells. It should also be noted that patients with favorable NKG2A GG and the unfavorable HLA-B-21 TT showed the highest LFS in the analysis. At first glance, this may seem contraintuitive, but these patients may benefit from having both highly educated KIR^+^ subsets (due to HLA-B-21 TT) and a larger subset of more functional NKG2A^+^ NK cells (due to NKG2A GG).

Multiple previous studies reported associations between the NKG2D SNP and clinical outcome in malignancy.^26–28 41^ For example, Hara *et al* observed that patients with chronic myeloid leukemia carrying NKG2D high natural cytotoxicity alleles achieved deeper molecular remission more quickly after receiving dasatinib.^27^ Similar results were reported in a survival analysis of different types of leukemia.^26^ The results presented in this study point to the possibility that such effects are instead driven by NKG2A variants. It should be underscored that our study does not preclude a functional impact of the gene variants on NKG2D. The favorable NKG2D G allele was associated with elevated expression of NKG2D,^18^ and high expression of activating receptors may be of importance for NK cell effector functions and clinical outcome in leukemia.^35 42^ However, in our combined regression analysis, the effect on LFS in AML was clearly associated with variations in NKG2A rather than NKG2D. It should also be noted that expression of both NKG2A and NKG2D is not restricted to NK cells and thus the impact of these polymorphisms may differ in different cell types and disease states.

Various NK cell-based cell therapy strategies are currently being evaluated for treatment of hematological malignancies and solid cancers.^43–45^ Much interest is directed toward transfusion of highly differentiated memory-like NK cells, but the field is still in its infancy.^45–48^ The findings in this study point toward a role also for more immature NKG2A-expressing NK cells. Notably, clinical trials using cytokine-induced memory-like (CIML) NK cells in AML have shown promising results.^49^ These NK cells have high expression of NKG2A, downregulated KIRs and exhibit enhanced functional responses against leukemic cells. In a limited number of patients, NKG2A expression was found to be a negative factor associated with treatment outcome.^50^ CIML NK cells have high proliferative capacity and prolonged persistence and are thus being evaluated as a source for development of chimeric antigen receptor (CAR) NK cells.^51^ Further studies are highly warranted, and the NKC locus gene variants described in this study could be a factor to consider in donor selection for CIML NK cell-based therapies.

In this study, all patients received HDC/IL-2 relapse-preventive immunotherapy. It would be interesting to determine whether NKG2A rs1983526 gene variants impact on clinical outcome of AML using other treatment modalities, but also in other hematological malignancies and solid cancers. As mentioned above, different adoptive NK cell therapies are gaining attraction in various hematological malignancies, and NKG2A blockade is currently being evaluated in a broad range of cancers.^52^ The net outcome of inhibitory receptor blockade in NK cells is difficult to predict as the intervention is not only blocking inhibitory signals at the NK cell—target cell encounter but also is potentially interfering with NK cell education, that is, reducing NK cell activity.^53^ Nevertheless, it will be interesting to see whether the NKG2A rs1983526 gene variant may be of predictive value in such immunotherapeutic interventions.

In conclusion, we report that the NKG2A rs1983526 skews the NK cell repertoire, affects NK cell function and is an independent prognostic marker for LFS in AML patients receiving HDC/IL-2 immunotherapy. Previous studies reporting associations between NKG2D variants and clinical findings may have overlooked the impact of the NKG2A variants. Further studies are thus warranted to address the role of these SNPs in cancer and related diseases.

## Data Availability

Data are available on reasonable request. For original data in the manuscript, please contact the corresponding author, fredrik.thoren@gu.se.
