# Supplementary material for: NKG2A gene variant predicts outcome of immunotherapy in AML and modulates the repertoire and function of NK cells
Source: J Immunother Cancer. 2023 Aug 30;11(8):e007202. doi: 10.1136/jitc-2023-007202 (PMC10471874; doi:10.1136/jitc-2023-007202)
Supplement: Supplementary data [file jitc-2023-007202supp001.pdf]

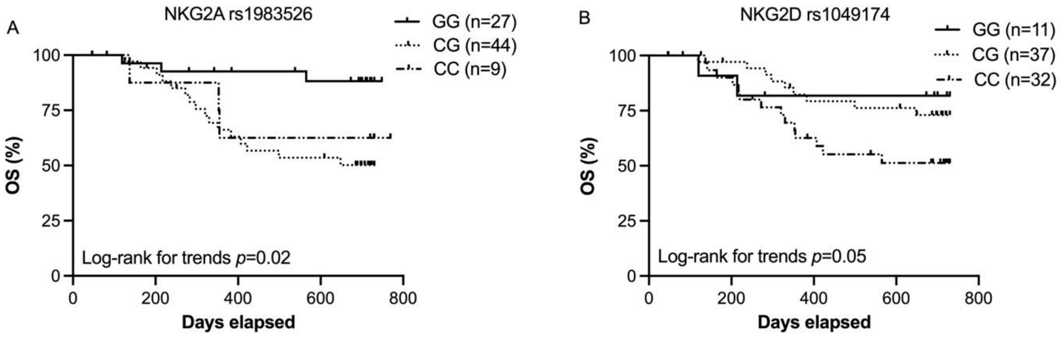

Supplemental Figure 1. Impact of NKG2A and NKG2D gene variants on outcome of immunotherapy in AML. (A) Overall survival of AML patients carrying NKG2A GG (n = 27), CG (n = 44) or CC (n=9) after receiving HDC/IL-2 treatment. (B) Overall survival of AML patients based on NKG2D GG (n = 11), CG (n = 37) or CC (n=32) after receiving HDC/IL-2 treatment.

Hussein BA, *et al. J Immunother Cancer* 2023; 11:e007202. doi: 10.1136/jitc-2023-007202

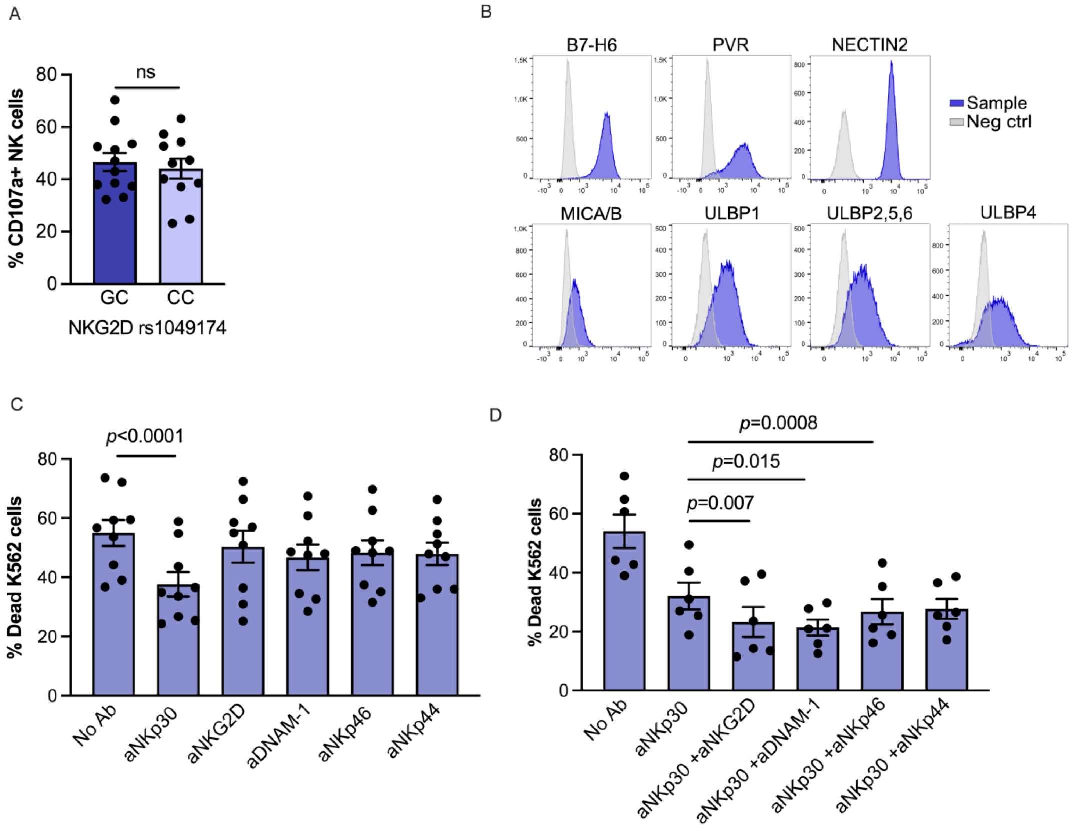

Supplemental Figure 2. Generation of an NKG2D-dependent model. (A) Degranulation assay of NK cells from healthy donors with NKG2D GC (n=12) or CC (n=11) pre-activated with IL-2 and cocultured with wild type K562. (B) Phenotype staining by flow cytometry of important NK cell receptor ligands on K562 wild type cells using fluorochrome-conjugated antibodies. (C-D) Cytotoxicity assay of healthy donor polyclonally activated NK cells against wild type K562 in presence and absence of different activating NK cell receptor blockade; single blockade (n=9) and anti-NKp30-combined blockade (n=6).

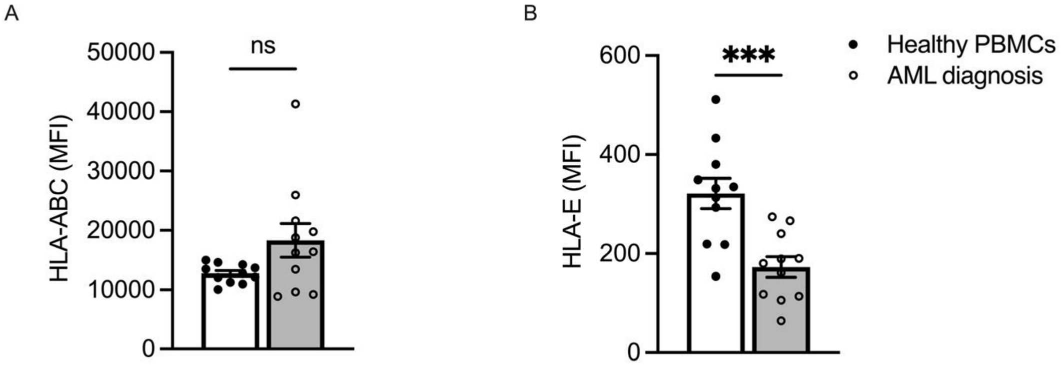

Supplemental Figure 3. Expression of MHC class I and HLA-E. (A) Expression of HLA-ABC and (B) HLA-E on PBMCs from healthy donors (n=11) and on CD34+ AML blasts (n=11).

Hussein BA, *et al. J Immunother Cancer* 2023; 11:e007202. doi: 10.1136/jitc-2023-007202

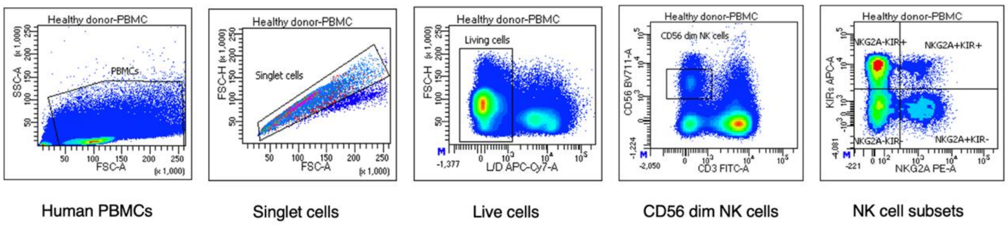

Supplemental Figure 4. Flow cytometry gating strategy to identify NK cell subsets.

Supplemental Table 1. Insert and crRNA sequences for cloning of plasmids needed for knock-out of ligand genes in target cell line. Primer sequences for PCR and qPCR.

Name	Sequence (5'→3')
Insert	tgctgtctcgagtaagtaataaacgttgcttaacgtctatcggtccgattcgtatta gctcggtcgattcgtcgagggcctatttcccatgattccttcatatttgcatatacga tacaaggctgttagagagataattggaattaatttgactgtaaacacaaagatatt agtacaaaatacgtgacgtagaaagtaataatttcttgggtagtttgcagttttaaa attatgttttaaaatggactatcatatgcttaccgtaacttgaaagtatttcgatttctt ggctttatatatcttgtggaaaggacgaaacaccgggtcttcgagaagacctgtttta gagctagaaatagcaagttaaaataaggctagtccgttatcaacttgaaaaagtgg caccgagtcggtgcggatccgtcgta
crRNA NCR3LG1	CACCAAGAGGCATTCCGACC
crRNA PVR	CCTGTTCGTCACGTTCCCGC
crRNA NECTIN2	GCGAGTTCAAGTGCTACCCG
PCR primer NCR3LG1_F	CCTCCAGATACCATCACACT
PCR primer NCR3LG1_R	AAGTGGAGCTCAAAGAGGTG
PCR primer PVR_F	CAAGTTGCAGACCATCCCAG
PCR primer PVR_R	GGGGGTCCTTACTCCCTGAG
PCR primer NECTIN2_F	CACACCTCGGAACATGAACA
PCR primer NECTIN2_R	GCGCCCAGCCAATACTTATT
qPCR primer NCR3LG1_F	GCTGACGACCGAAGGTGAT
qPCR primer NCR3LG1_R	GTGATGTTGAGGGGTTGGGA
qPCR primer PVR_F	GTGGACGGCAAGAATGTGAC
qPCR primer PVR_R	ATCATAGCCAGAGATGGATACCT
qPCR primer NECTIN2_F	GAGGACGAGGGCAACTACAC
qPCR primer NECTIN2_R	TGGTTCTTGGGCTTGGCTAT
qPCR primer MICA/B_F	AAGGACCAGAAAGGAGGCTT
qPCR primer MICA/B_R	TCCCCATCGTAGTAGAAATGC
qPCR primer ULBP1_F	GGCAGATGAGGAGAGTTGTTT
qPCR primer ULBP1_R	AGGACCCAGACCAGGCTAAC
qPCR primer ULBP2_F	CGCTACCAAGATCCTTCTGTG
qPCR primer ULBP2_R	AAGAGAGTGAGGGTCGGC
qPCR primer ULBP3_F	CGCTCCTGGTCTACAATGGC
qPCR primer ULBP3_R	TCTGGGCAAATGAATGATGGTG
qPCR primer CD48_F	GAGCAGCAAGAATGGCACG
qPCR primer CD48_R	TCATCTCAGGTAAGTAACAGGC
qPCR primer CD58_F	GTTCTTTCTTTATGTGCTTGAGTCT
qPCR primer CD58_R	TGGCTGTTGTAATGCTCTGGT
qPCR primer CLEC2B_F	CCACTCAACATGCCGACCTA
qPCR primer CLEC2B_R	TGCCATCTTCAGTCCAATCCA
qPCR primer BAG6_F	CACCCAACCATCCTTCCCCT
qPCR primer BAG6_R	CACCCAGAACCTCGTAGTAGC

BMJ Publishing Group Limited (BMJ) disclaims all liability and responsibility arising from any reliance placed on this supplemental material which has been supplied by the author(s)

Supplemental Table 2. List of flowcytometry markers

Marker	Fluorophore	Clone	Manufacturer
CD107a	BUV395	H4A3	BD Bioscience
NKG2D	Unconjugated	BAT221	Miltenyi Biotec
CD3	PerCP-Cy5.5	SK7	BD Bioscience
CD56	BV711	NCAM 16.2	BD Bioscience
KIR2DL1/2	APC	NKVFS-1	Miltenyi Biotec
KIRD3DL1/2	APC	REA970	Miltenyi Biotec
NKG2A	PE	Z199	Beckman Coulter
NKp44	Unconjugated	KS38	Kindly provided by Prof. Silvia Parolini, Brescia, Italy
NKp46	Unconjugated	KL247	Kindly provided by Prof. Silvia Parolini, Brescia, Italy
NKp30	Unconjugated	F252	Kindly provided by Dr. Daniela Pende, Genoa, Italy
DNAM-1	Unconjugated	F5	Kindly provided by Dr. Daniela Pende, Genoa, Italy
IFNγ	BUV395	B27	BD Bioscience
Granzyme B	BV421	GB11	BD Bioscience
B7-H6	Unconjugated	17B1.3	Kindly provided by Prof. Eric Vivier, Marseille, France
PVR	Unconjugated	5A10	Kindly provided by Moretta lab, Genoa, Italy
Nectin-2	Unconjugated	L14	Kindly provided by Moretta lab, Genoa, Italy
MICA/B	Unconjugated	6D4	Miltenyi Biotec
ULBP1	Unconjugated	170818	R&D Systems
ULBP2,5,6	Unconjugated	165903	R&D Systems
ULBP4	Unconjugated	6E6	Santa Crus Biotechnology
Live/Dead near IR	NA	NA	Invitrogen
CellTrace Violet	NA	NA	Invitrogen

Hussein BA, *et al. J Immunother Cancer* 2023; 11:e007202. doi: 10.1136/jitc-2023-007202
